# Structures and thermodynamics of water encapsulated by graphene

**DOI:** 10.1038/s41598-017-02582-7

**Published:** 2017-06-01

**Authors:** Shuping Jiao, Chuanhua Duan, Zhiping Xu

**Affiliations:** 1 Applied Mechanics Laboratory, Department of Engineering Mechanics, and Center for Nano and Micro Mechanics, Tsinghua University, Beijing, 100084 China; 2 Department of Mechanical Engineering, Boston University, Boston, MA 02215 USA

## Abstract

Understanding phase behaviors of nanoconfined water has driven notable research interests recently. In this work, we examine water encapsulated under a graphene cover that offers an ideal testbed to explore its molecular structures and thermodynamics. We find layered water structures for up to ~1000 trapped water molecules, which is stabilized by the spatial confinement and pressure induced by interfacial adhesion. For monolayer encapsulations, we identify representative two-dimensional crystalline lattices as well as defects therein. Free energy analysis shows that the structural orders with low entropy are compensated by high formation energies due to the pressurized confinement. There exists an order-to-disorder transition for this condensed phase at ~480–490 K, with a sharp reduction in the number of hydrogen bonds and increase in the entropy. Fast diffusion of the encapsulated water demonstrates anomalous temperature dependence, indicating the solid-to-fluid nature of this structural transition. These findings offer fundamental understandings of the encapsulated water that can be used as a pressurized cell with trapped molecular species, and provide guidance for practical applications with its presence, for example, in the design of nanodevices and nanoconfined reactive cells.

## Introduction

The formation mechanism of ordered and disordered hydrogen-bond (H-bond) networks has been a unsolved puzzle in understanding the structures and behaviors of water and ice^1^. Although significant attention has been paid to elucidate the phase diagram of bulk water in the temperature-pressure spaces, characterization of nanoconfined water at room temperature has only started recently. For example, water in chain and tubular forms are found inside carbon nanotubes^2^. Two-dimensional (2D) ordered H-Bond networks with various topologies are observed on solid surfaces^3^ or within the nanoscale capillary galleries in layered materials^4^. The formation and stabilization of these ordered phases of water were proposed to benefit from the effects of nanoconfinement, lattice matching, surface interaction, and pressure^5–7^. This much enriched phase diagram of water in a confined space not only introduces new forms and thermodynamic behaviors of water, such as the 2D ice and fast mass transport in hydrophobic nanochannels^4^, but also poses critical questions on the fundamental understanding of nanoconfined water.

As a finite system, the surface of small water droplets or clusters plays an important role in defining their thermodynamics^8^. Similarly, under nanoconfinement, the interface between water and structures not only modulates the H-bond network near the interface, leading to structural ordering such as layering or crystallization, but also applies an anisotropic pressure onto the water condensation. For example, in-plane pressure on the order of 1 GPa was estimated for water capillary confined between graphene oxide layers, although the out-of-plane can be absent in a relaxed structure^4, 7, 9^. In the development of nanoelectronic devices with thin films deposited onto substrates, intercalated water has been characterized at the interface^10–14^. Encapsulation by wrinkles or ripples in the graphene membrane thus provides an isolated, strong nanoconfinement for water, which could then be used as a test chamber to probe the thermodynamics of water in this specific condition. Graphene encapsulation has also been proposed as a reactive cell to explore molecular processes within the trapped molecules, liquids or solids^15, 16^. Moreover, the encapsulated confinement is different from those in nanoslits, nanopores or on surfaces because of the presence of pressure in encapsulated water (EW) resulted from the adhesion between graphene and the substrate^17^, which thus adds new understandings to those on the existing models of nanoconfined water.

In this work, we explore the structures and thermodynamics of water encapsulated by a graphene layer deposited on a solid surface by performing molecular dynamics (MD) simulations. We first report the ordered and disordered molecular structures of water condensations in the encapsulation, and then explore their structural transitions and thermodynamics based on free energy analysis. We also analyze molecular and collective diffusion of EWs that demonstrates a strong correlation with their molecular structures.

## Results

### Structures of ordered and disordered water

**Figure 1:**
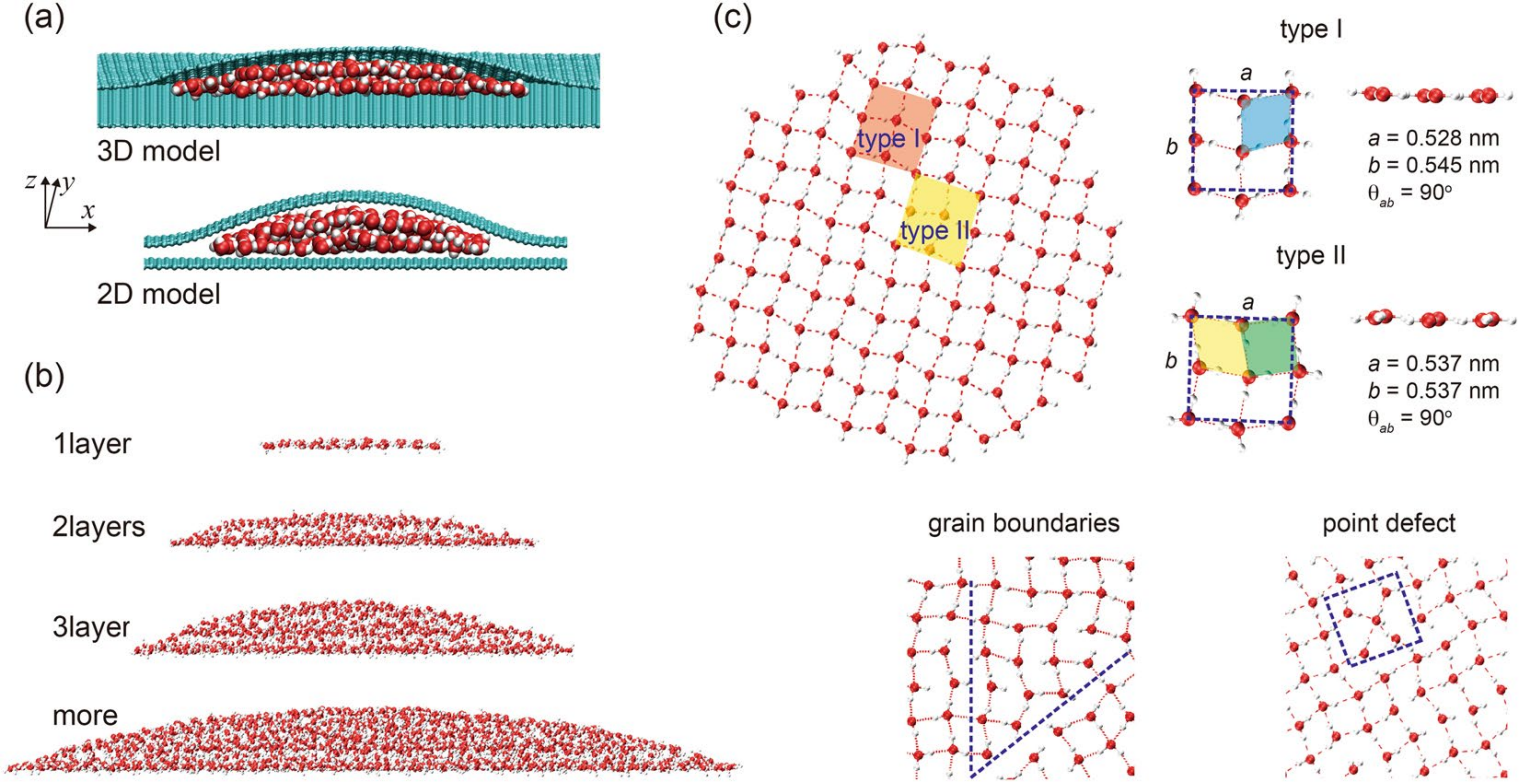
(**a**) Cross-section simulation snapshots of 3D (hemispheric) and 2D (half-cylindrical) models for water encapsulation under graphene. (**b**) Mono-, bi- and tri-layer water structures in the 3D model, with the number of water molecules *N*_W_ = 115, 546, 986 at 300 K, which disappear with *N*_W_ > ~1000. (**c**) Structures of monolayer water showing ordered structures of two different lattice types, as well as defects (grain boundaries and point defects). The lattice constants are denoted.

**Figure 2:**
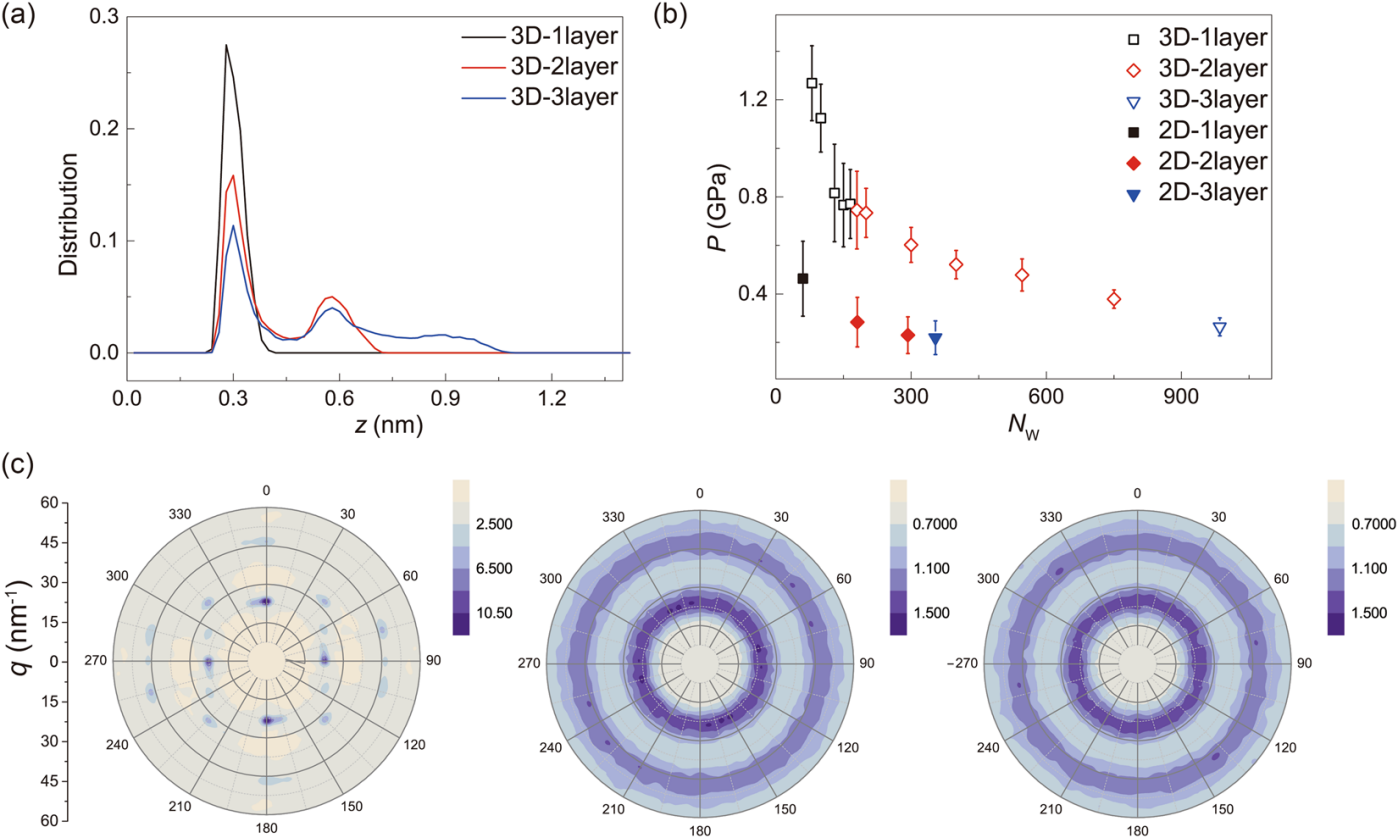
(**a**) Density profiles at 300 K, measured along the *z* direction of encapsulated water in the 3D model, with *N*_W_ = 115, 546 and 986 for 1, 2 and 3 layers. (**b**) Pressure in the water condensation plotted as a function of the number of water molecules at *T* = 300 K. (**c**) In-plane structure factors of nanoconfined mono-, bi- and tri-layer water with *N*_W_ = 115, 546, 986 at *T* = 300 K. The error bars are obtained from the thermodynamic fluctuations measured in MD simulations. Here the wave vector **q** is represented as its amplitude *q* and orientation in the polar plots. The color denotes the amplitude of structure factor *S*(**q**).

To model the nanoconfined water, we encapsulate a water droplet between two graphene sheets, where the bottom layer is fixed, representing a solid substrate. The top layer covers the droplet and is free to deform, to accommodate the structural changes in water. Models are constructed with three-dimensional (3D, hemispheric) and 2D (half-cylindrical) encapsulation, respectively (Fig. 1a). The atomic structures are equilibrated in our MD simulations at specific temperature. The simulation results show that at room temperature, ordered structures form in the encapsulation. Layered structures are distinct for mono-, bi- and tri-layers but not for larger droplet with the number of water molecules *N*_W_ beyond ~1000 (Fig. 1b). For our 3D models, the transition from mono-layer to bi-layer structures occurs at *N*_W_ = ~165–180, and the latter structure further changes into tri-layer EW with *N*_W_ increases to ~750–986. It should be noticed that the critical number of water molecules depends on the temperature as well (Figure S1). The density profiles also characterize weakened layering order when the size of EW increases from mono-layer to the bi- and tri-layers, demonstrating the increasing fluidity (Figs 2a and S2).

The presence of interlayered H-bonds within the EW and reduced pressure weaken the layered order (Figs 2b and S2). This effect is similar to the observations for water confined between planar surfaces, where the layered order is reduced with increasing interlayer distance, although the non-flat confining boundaries in our models results in less ordered structures^7^. Moreover, in the thermal equilibrium at room temperature, molecules in the monolayer EW are able to vibrate and librate in the H-bond network, but molecular rotation and diffusion within the encapsulation is not activated, indicating the nature of solidity, while in either bi- or tri-layers both rotation and diffusion are observed and the intralayer diffusion is more prominent than that across the layers.

In addition to the layering order, in-plane molecular structures with regular H-bond network are also identified. For the water monolayer encapsulated at room temperature, we find ordered region with almost-perfect 2D lattices, disordered region between them that include grain boundaries, as well as point defects (Fig. 1c). The H-bonds align with the plane of water layers in general. Each of the water molecules in these lattice bonds with neighboring molecules through four H-bonds, following the ice rule^1^. Structure factor analysis demonstrates a distinct near-square symmetry for monolayer EW, which is absent in the bi- and tri-layers (Figs 1c and 2c), and this conclusion holds for the 2D encapsulations as well. Bond length and angle analysis of the H-bonding network show an average H-bond length of *l*_O-O_ ~0.278 nm, and the bond angles span over a wide region around *θ*_O-H-O_ ~160°.

In the crystalline domains, we identify two types of 2D lattices, which are shown in Fig. 1c. Several types of quasi-2D ice structures, including rhombic structures, have been observed recently between hydrophobic walls with the interlayer distance *d* < 0.7 nm^18, 19^. The structures characterized are mostly the same or similar (with different out-of-plane or lateral structures, depending on the model used) as the type II structure identified in this work although the confinement is quite different^18, 19^, while type I has not been reported in these studies. The change in nanoconfining conditions also leads to different phase diagrams of water, which depends on the water models used in the simulations as well. Considering the wide appearance of EWs in the graphene-substrate setup, our findings here have significant practical implications in the design of nanoelectronics. The two lattices reported here both have four water molecules in the unit cell. The difference exists in that for type I, there are four almost-identical rhombuses, and while the lattice type II contains two squares and two rhombuses. These two types of lattices are also indicated by three peaks at about 75°, 90°, 105° in the distribution of O-O-O angles in our structural analysis. It is interesting that these two types of lattice are geometrically compatible and can co-exist, as we observed in the simulation snapshots. The mixing nature of type I and II in our MD simulations and presence of defects in the ordered H-bond network originate from the incompatible spatial confinement and energy barriers that prohibit further perfection of the defective structures into a single crystal domain.

### Thermodynamics of encapsulated water

These evidences of ordered water structures inside graphene encapsulation at room temperature are interesting not only because its formation mechanism offers hints in the understanding of water, but also provides an ideal platform to explore thermodynamics of condensed matter in a nanoconfined space. When a graphene sheet is deposited onto the substrate, the competition between graphene elasticity and the substrate interaction results in an optimal conformation of the EW^20^. Adhesion between two graphene layers further leads to a pressure inside the encapsulation. From our MD simulation results, we find that the pressure is on the order of *P* = 0.1–1 GPa, which decreases with the number of water molecules enclosed (Fig. 2b)^7^. Here the pressure within EW is averaged over the atomic pressure tensor of all water molecules, which includes both kinetic energy and virial contributions and is anisotropic in this situation^21^. Consequently, it contains water-water and carbon-water interactions but not the carbon-carbon interaction in evaluation, and could be decomposed into in-plane and vertical components. It should be pointed out that, unlike the pressure in simple fluids with pairwise interactions, pressure in the liquid/solid cell under the graphene cover presents the anisotropy, demonstrating the structured nature of nanoscale EW. In our work, the amplitude of in-plane pressure is usually about half of the normal pressure. Considering the ultrahigh in-plane tensile stiffness of graphene *k* = ~340–690 N/m and low bending stiffness of *D* = ~1 eV^22^, this pressure will bend the graphene sheet instead of stretching it, adapting to the deformation of EW. Our structural analysis supports this conclusion by showing that the bond length in graphene is almost identical to that in the undeformed structure. From the phase diagram of bulk water, we find its mass density *ρ*_0_ is ~1.1–1.2 g/cm^3^ at pressure *P* = ~0.3–1 GPa^1^, which aligns with our simulation results for EWs, *ρ* = ~1.07–1.25 g/cm^3^, although the H-bond network in the EW is different from that in the bulk.

**Figure 3:**
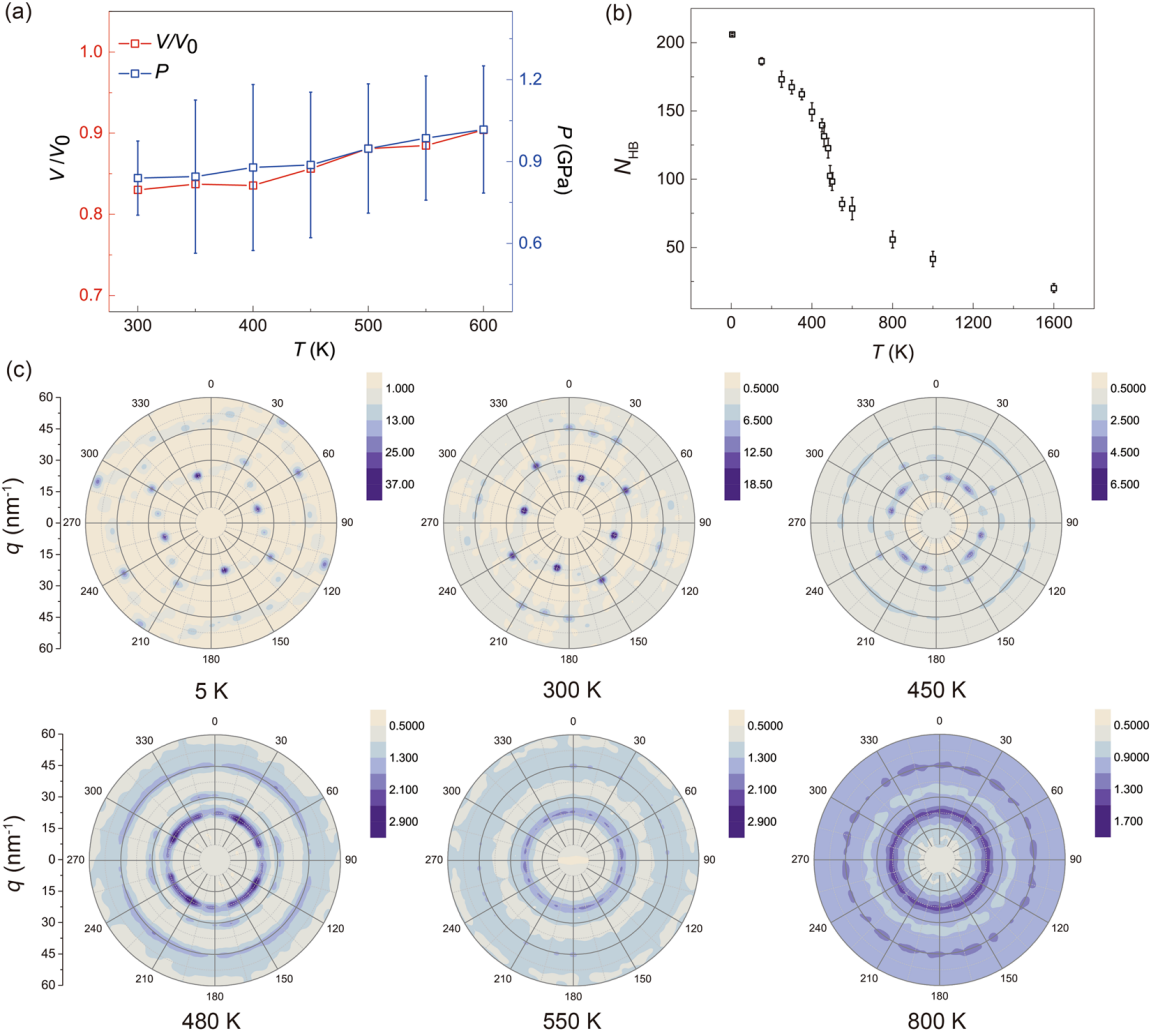
(**a**) Pressure, volume changes as function of *T*, where *V*_0_ is volume of bulk water at standard atomosphere, for *N*_W_ = 115 in the 3D EW model. (**b**) Number of hydrogen bonds, *N*_HB_, in the EW, plotted as a function of temperature. The error bars measure the thermodynamic fluctuations. (**c**) Structure factors of monolayer EW, demonstrating a solid-to-fluid phase transition at ~480–490 K.

The phase stability of ordered water structures is assessed here by performing MD simulations within a wide range of temperature. We find that the EW is stable up to the condition where the graphene structure becomes unstable. In this work, we limit our discussion within the temperature range of *T* = 200–600 K for the interest of practical applications in nanoelectronic devices. We find that, as *T* increases, the pressure gently increases from 0.81 to 1.0 GPa for a monolayer EW with 115 water molecules, and the molecular volume increases slightly from 0.83*V*_0_ to 0.9*V*_0_. Here *V*_0_ is the molecular volume in bulk water at standard atomosphere (Fig. 3a). However, there is distinct reduction in the number of H-bonds at 480–490 K, implying a pronounced structural change in the EW (Figs 3b,c and S3).

**Figure 4:**
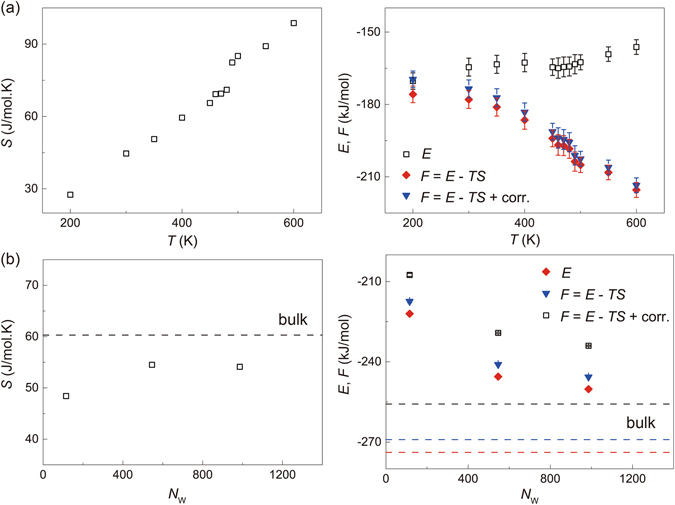
Entropy (*S*), total energy calculated from MD simulations (*E*), free energy (*F*) for the EW as temperature *T* = 200–600 K (**a**) and water number *N*_W_ = 115, 546, 986 (mono-, bi-, tri-layer), as well as bulk water (**b**). The correction on free energy with ZPE and heat capacity terms are considered, using the 2PT model. The error bars measure the energy fluctuations in MD simulations.

To obtain more insights into the thermodynamics of EW, we calculate the entropy based on the two-phase thermodynamics (2PT) model developed by Lin *et al*.^23^, as well as the free energy *F* = *E* − *TS* with additional correction terms for the zero point energy (ZPE) and heat capacity. The entropy shows discontinuous jump near *T* = 480–490 K, signaling a first-order phase transition (Fig. 4a), which agrees with the observed breakdown of ordered H-bond network (Fig. 3b). We also find that the EW has much lower entropy (*S* = 48.4, 54.4 and 54.1 J/mol.K for mono-, bi- and tri-layers) than that of the bulk water (*S*_0_ = 60.3 J/mol.K), which corresponds to high -*TS* values in the free energy that must be compensated by the reduction in the total energy, *E*, of EW (Fig. 4b). Specifically, the entropy of monolayer EW is comparable, and slightly lower than that of the intercalated water in the slit between planar graphene sheets, while the total energy and free energy are much lower (Table 1). As *T* increases from 200 to 600 K, the change in *E* is much smaller than that in the free energy *F*, which keeps decreasing (Fig. 4b). The distinct changes in structures of monolayer EWs can also be characterized by the structure factor *S*(**q**) summarized from the MD simulations of 3D and 2D structure.

**Table 1:** Thermodynamic properties (entropy *S*, total energy *E* and free energy *F*) at 300 K calculated for each water molecule under different confinements.

	*S* (J/mol.K)	*E* (kJ/mol)	*F* (kJ/mol)
Partial intercalation*	51.02	−90.34	−105.64
Full intercalation*	49.93	−195.90	−210.88
Encapsulated water	48.40	−207.58	−222.10

*For full intercalated water monolayer between graphene sheets, additional constraints come from the periodic boundary conditions applied in the lateral dimensions. Models are constructed with an interlayer distance of 0.64 nm.

## Discussion

### Understanding structural ordering of encapsulated water

From our MD simulation results, we conclude that the formation of ordered structures in the EW arises from the pressure induced by encapsulation and the nanoscale confinement where the lattice of graphene (both the cover and substrate) offers a quasi-epitaxial template for EW. To elucidate the dominating mechanism in the formation of 2D ‘ice’ structures, a simulation of intercalated water between two rigid parallel graphene sheets is also carried out. We find that a single layer 2D ‘ice’ forms even in the absence of pressure, with an interlayer distance *d* = 0.64 nm (Figure S4). However, the in-plane order is lower than that in the EWs, as indicated by the structure factor analysis. We also notice that, the pressure *P* = ~0.22–0.46 GPa in the 2D encapsulation is lower than that in the 3D encapsulation due to the weaker confinement, and as a result, the EW structure is less ordered in the 2D. We further tune the interaction strength between water molecules and graphene, which adjusts the pressure in the nanoconfined water. The simulation results show that the in-plane lattice structures vanish as the van der Waals interaction decreases, where the confinement and pressure become weaker and lower. A 20% enhancemnt of the interaction strength does not lead to noticeable change in the water structures and critical temperature for the solid-liquid transition (Figure S5). Considering the wide range of water contact angle values reported in the literature, our parametric study shows the dependence of our results on the nature of water-graphene interaction that has not yet been clarified now. Moreover, our additional simulation results for water encapsulated between graphene and three different substrates (silica and hexagonal boron-nitride (h-BN), and Cu (111) surfaces) show that ordered 2D lattice of water molecules appears on h-BN, Cu(111) but not for silica (Figure S6). This finding could be attributed to the perturbation of silica on the in-plane H-bond network, due to the participation of surface hydroxyl groups in it. From these facts, we conclude that the nanoconfinement is critical for the formation of layered water structures, while structure of the H-bond network controls the in-plane order for the room-temperature 2D ice.

### Dynamical behaviors of encapsulated water

**Figure 5:**
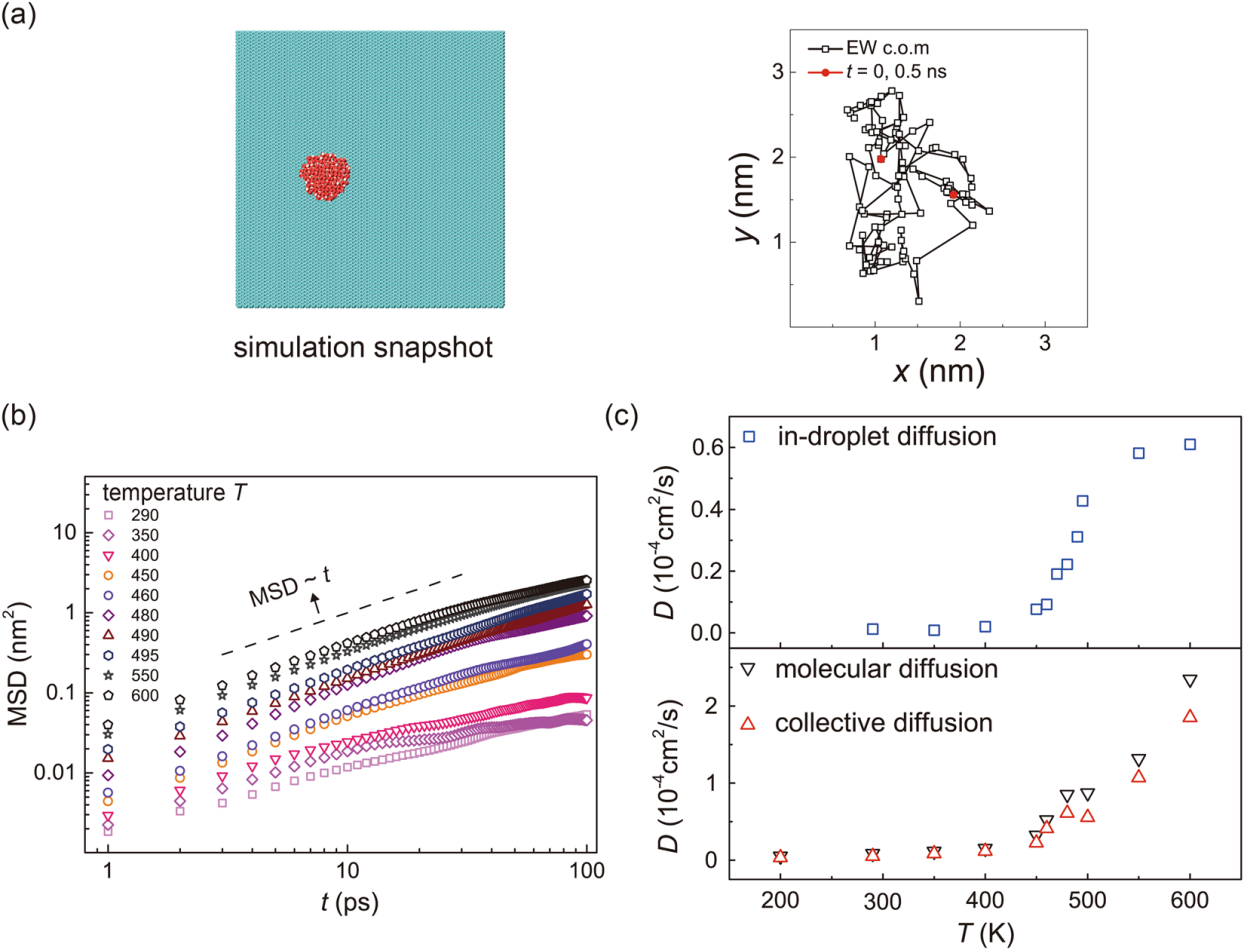
(**a**) A simulation snapshot and a 0.5 ns-long center-of-mass trajectory of the encapsulated water with *N*_W_ = 115, at *T* = 300 K. Here only the graphene substrate is shown. (**b**) MSD of water molecular diffusion in a monolayer EW, calculated at different temperature. (**c**) Diffusion coefficient *D* calculated for both molecular diffusion and the collective diffusion of EW. To calculate the in-droplet diffusion, the collective motion is subtracted from molecular diffusion.

The phase transition at 480–490 K is likely to be a solid-to-fluid transition in 2D based on our structural analysis. To further characterize this transition, we explore the dynamical behaviors of water molecules in EW by computing the mean square distance (MSD) of molecular diffusion from trajectories of the molecules or collective diffusion from the center-of-mass motion of EWs (Fig. 5a). The graphene substrate is constrained in the MD simulations here, and externals force corresponding to the constraint preserves the conservation of total momentum while large-amplitude displacement is allowed for the water structures. The results clearly demonstrate the appearance of fluidity above the transition temperature. Dramatic increase of the MSDs occurs in the temperature range of ~480–490 K, which indicates a phase transition. More interestingly, we find that the EWs could diffuse collectively under the graphene coating, mostly in translational motion and gentle libration. From the data of MSDs, although a sub-diffusive behavior is identified, we calculate the diffusion coefficient *D* of the collective motion of EW (Fig. 5c), which is on the order of 10^−5^ cm^2^/s at room temperature. This fast diffusion of high-pressure water cells indicates an efficient way to transport nanoscale water at interfaces with two-dimensional materials. However, the value of *D* for water diffusion within the droplet, where the collective motion is subtracted from molecular diffusion, is only ~10^−7^ cm^2^/s at 200–400 K and ~10^−6^ cm^2^/s at 450 K (Fig. 5c), which is significantly lower than that in the bulk water at ambient condition (*D*_0_ = ~10^−5^ cm^2^/s)^2, 5^. This result confirms the solid-like behavior of EWs at the room temperature, and the occurrence of solid-to-fluid transition at ~480–490 K.

### Assessment of water models

In MD simulations, the structures and thermodynamics of water predicted rely on the potential model used. Especially in the EW, the flexibility of water models could be essential as the pressure therein is high (~0.1 GPa)^24, 25^. Although there is no ‘perfect’ model that could capture all the complex behaviors of water, we verify our findings by performing comparative studies using the rigid SPC/E, TIP4P/2005 models and the flexible TIP4P/2005f model that are widely used for nanoconfined water and other forms of water^7, 26–28^. The results lead to the same conclusion in general. The water structures predicted are the same, and the transition temperature predicted is almost the same (~480–490 K) from TIP4P/Ew, TIP4P/2005 and TIP4P/2005f models, although the range of transition predicted from the flexible model (TIP4P/2005f) is broader (~470–510 K). The value obtained by using the SPC/E model is slightly lower (*T* = ~400 K) than the others, although the phase transition behaviors are similar. Consequently, the results presented in this work based on the TIP4P/Ew model offers a reliable prediction.

## Conclusion

In brief, we explored the atomic structure and thermodynamics of encapsulated water covered by a graphene sheet. Our MD simulation results show that the formation mechanism of layered structures is mainly attribute to the spatial confinement, where the graphene sheets offer an ‘epitaxial’ template, and the encapsulating pressure is critical for the appearance of in-plane orders. A first-order, solid-to-fluid phase transition in 2D is identified for the encapsulated water, which is characterized by a discontinuous change in the entropy and structure of the H-bond network, as well as an anomalous temperature dependence of the diffusion behavior. The wide temperature window of stable nanoconfined water structures under the cover of graphene sheet offers an ideal platform to explore the intriguing behaviors of water in a confined environment, and our findings here provide some fundamental understandings of it.

We notice a number of recent studies that report similar 2D ice structures of water confined between two parallel graphene sheets, which align with our type I and type II structures although the condition of confinement is different from our model^29–32^. The robustness of these 2D lattices highlights their significance for both theoretical interests and practical applications in nanoscale material and device design. For example, trapped water between 2D materials and their substrate is common in device development^13–15^, graphene encapsulation is also ideal for coating and trapping volatile molecules, liquids or solids for microscopy studies for it is conductive, chemically inert, impermeable and atomically conforms to most substrates^15, 16^. Consequently, our structural and thermodynamics analysis of the EWs, as well as discussion on their molecular and collective diffusion add more understandings to these unique phases of water.

## Methods

### Molecular structures

Both hemispheric models with the number of water molecule *N*_W_ = 80–1536 and half-cylindrical models with *N*_W_ = 60–708 are constructed in this work. In the 3D (hemispheric) model, the lateral size of the graphene sheets is 20 nm × 20 nm, and an open boundary is used in the *z* direction. In the 2D (half-cylindrical) model, the size of graphene sheet is 25 nm × 2.13 nm, and a periodic boundary condition (PBC) is applied only in the *y* direction. No PBCs are applied in other dimensions (*x* and *y* in the 3D model and *x* in the 2D model). To construct the encapsulated systen, a graphene sheet is directly deposited on the substrate, with a water droplet pre-equilibrated on it.

### Molecule dynamics simulations

We perform molecular dynamics simulations using the large-scale atomic/molecular massively parallel simulator (LAMMPS)^21^. The all-atom optimized potentials for liquid simulations (OPLS-AA) are used for graphene, which predicts a Young’s modulus of 843.5 GPa and a bending stiffness of 1.77 eV, both are consistent with the values reported in the litearture^9, 33–35^. TIP4P/Ew and the extended simple point charge model (SPC/E) models of water are used for a comparative study, which were both widely adopted for MD simulations of water and its phase transition behaviors^36–38^. Our MD simulation results show that these two models predict the same water structure, but slightly different structural transition temperature. The interaction between graphene and water is described with the set of simulation parameters, *ε*_C-O_ = 4.063 meV, *σ*_C-O_ = 0.319 nm, and a cut-off is made at 1.2 nm, which predicts a water contact angle (WCA) of *θ*_c,G_ = 98.4° for graphene that is in consistency with experimental measurements^35^. The value of *ε*_C-O_ is modified to probe the dependence of our prediction on the strength of water-graphene interaction that is still under debate in the literature^39–41^, as well as the consideration of the substrate underneath graphene^42, 43^. The Lennard-Jones and atomic-charge parameters used for crystalline silica, hexagonal boron nitride and Cu (111) surfaces are summarized in Table S1. The long-range Coulomb interactions are computed by using the particle-particle particle-mesh algorithm (PPPM)^44^. The time step for the equation-of-motion integration is 1 fs, with the SHAKE algorithm applied for the stretching terms between oxygen and hydrogen atoms of water to reduce high-frequency vibrations that require a very short time step. Both the 3D and 2D structures are equilibrated using a Berendsen thermostat at 200–600 K for 2 ns, with a damping constant *τ*_T_ = 100 fs. It should be noted here that although MD simulations are known to suffer from hysteresis for modeling solid-liquid phase transition in homogeneous systems, the structure of finite-size water encapsulation studied in this work is sufficiently heterogeneous to exclude such metastability, which is validated by the alignment between simulated structures obtained by rising and lowering the temperature, respectively. MD simulations with temperature up to *T* = 1600 K are performed to explore the thermal stability of EWs. For the thermodynamics analysis of entropy and free energy, we carry out equilibrium MD simulations in a NVT ensemble using the Nosé-hoover thermostat for another 0.2 ns, where the MD trajectories are written out every 4 fs for 50 ps^23^. In exploring the phase behaviors at different temperatures, equilibration simulations are carried out for more than 1 ns, which is sufficiently long by tracking the evolution of temperature profile. The atomic pressure tensor is calculated from the kinetic and virial parts^45^.

### Structural analysis

To analyze the H- bond network in the EW, we choose a geometry-based criterion for its definition^46, 47^. For two water molecules are linked by one H-bond when three conditions are fulfilled: (1) the distance between the oxygens of both molecules is smaller than 3.6 Å, (2) the distance between the oxygen of the acceptor molecule and the hydrogen of the donor is smaller than 2.45 Å, (3) the angle defined within the dimer geometry is smaller than 30°.

The in-plane structure factors of layered EWs, by considering the oxygen atoms as the lattice sites, is calculated as^37, 48^

1 $$S({\bf{q}})=\langle \frac{1}{{N}_{{\rm{W}}}}[{(\sum _{i=1}^{{N}_{{\rm{W}}}}\cos ({\bf{q}}{\boldsymbol{.}}{{\bf{r}}}_{i}))}^{2}+{(\sum _{i=1}^{{N}_{{\rm{W}}}}\sin ({\bf{q}}{\boldsymbol{.}}{{\bf{r}}}_{i}))}^{2}]\rangle $$

here **r**_j_ is position of the *j*-th oxygen atom in the plane, *N*_W_ is the number of water molecules, and **q** is wavevector. <…> denotes time average in thermal equilibrium.

### Characterization of molecular and collective diffusion

The diffusion coefficient *D* is calculated from the molecular trajectories of water by using the Einstein’s definition relating the correlation function of atomic positions **r**_*i*_, or the mean-square distance (MSD), to the diffusivity *D* = lim_*t*−>∞_ < |**r**(t) − **r**(0)|^2^ >/2*d*_i_ *t*. Here *d*_*i*_ is the dimension of space, *t* is the simulation time, and <…> is the ensemble average. The collective motion of EW is calculated from its center-of-mass trajectories, and fort he monolayer EWs, there is only in-plane diffusion with *d*_i_ = 2. In our simulations with a time span of a few nanoseconds, the MSD <|**r**(*t*) − **r**(0)|^2^> is calculated based on the time-series of all oxygen atom position. The whole set of trajetory (0–100 ps) is analyzed, with 1500 time-averages starting from different time point in the series.

## Electronic supplementary material

Supplementary Information Material

## References

[1] Hobbs, P. V. *Ice Physics* (Oxford University Press, 1974).

[2] Koga K, Gao GT, Tanaka H, Zeng XC (2001). Formation of ordered ice nanotubes inside carbon nanotubes. Nature.

[3] Hu J, Xiao X-D, Ogletree DF, Salmeron M (1995). Imaging the condensation and evaporation of molecularly thin films of water with nanometer resolution. Science.

[4] Nair RR, Wu HA, Jayaram PN, Grigorieva IV, Geim AK (2012). Unimpeded permeation of water through helium-leak-tight graphene-based membranes. Science.

[5] Han S, Choi MY, Kumar P, Stanley HE (2010). Phase transitions in confined water nanofilms. Nat. Phys.

[6] Carrasco J, Hodgson A, Michaelides A (2012). A molecular perspective of water at metal interfaces. Nat. Mater..

[7] Algara-Siller G (2015). Square ice in graphene nanocapillaries. Nature.

[8] Hill, T. L. *Thermodynamics of Small Systems Part I & II* Dover Publications, (1994).

[9] Jorgensen WL, Maxwell DS, Tirado-Rives J (1996). Development and testing of the OPLS all-atom force field on conformational energetics and properties of organic liquids. J. Am. Chem. Soc..

[10] Shim J (2012). Water-gated charge doping of graphene induced by mica substrates. Nano Lett..

[11] Lee D, Ahn G, Ryu S (2014). Two-dimensional water diffusion at a graphene-silica interface. J. Am. Chem. Soc..

[12] Severin N, Lange P, Sokolov IM, Rabe JP (2012). Reversible dewetting of a molecularly thin fluid water film in a soft graphene–mica slit pore. Nano Lett..

[13] Olson EJ (2015). Capacitive sensing of intercalated H_2_O molecules using graphene. ACS Appl. Mater. Interf..

[14] Wang Y, Xu Z (2016). Water intercalation for seamless, electrically insulating, and thermally transparent interfaces. ACS Appl. Mater. Interf..

[15] Lee MJ (2012). Characteristics and effects of diffused water between graphene and a SiO_2_ substrate. Nano Res.

[16] Yuk JM (2012). High-resolution EM of colloidal nanocrystal growth using graphene liquid cells. Science.

[17] Li Q, Song J, Besenbacher F, Dong M (2015). Two-dimensional material confined water. Acc. Chem. Res..

[18] Zhao W-H (2014). Highly confined water: Two-dimensional ice, amorphous ice, and clathrate hydrates. Acc. Chem. Res..

[19] Zhao W-H, Bai J, Yuan L-F, Yang J, Zeng XC (2014). Ferroelectric hexagonal and rhombic monolayer ice phases. Chem. Sci.

[20] Zhu W (2012). Structure and electronic transport in graphene wrinkles. Nano Lett..

[21] Plimpton S (1995). Fast parallel algorithms for short-range molecular dynamics. J. Comp. Phys.

[22] Lee C, Wei X, Kysar JW, Hone J (2008). Measurement of the elastic properties and intrinsic strength of monolayer graphene. Science.

[23] Lin ST, Blanco M, Goddard WA (2003). The two-phase model for calculating thermodynamic properties of liquids from molecular dynamics: Validation for the phase diagram of Lennard-Jones fluids. J. Chem. Phys..

[24] Vega C, Abascal JLF, Sanz E, MacDowell LG, McBride C (2005). Can simple models describe the phase diagram of water?. J. Phys.: Condens. Matter.

[25] L MW, Franck EU (1981). Ion product of water substance, 0–1000 °C, 1–10,000 bars new international formulation and its background. J. Phys. Chem. Ref. Data.

[26] Berendsen HJC, Grigera JR, Straatsman TP (1987). The missing term in effective pair potentials. J. Phys. Chem..

[27] Abascal JLF, Vega C (2005). A general purpose model for the condensed phases of water: TIP4P/2005. J. Chem. Phys..

[28] González MA, Abascal JLF (2011). A flexible model for water based on TIP4P/2005. J. Chem. Phys..

[29] Zhu Y, Wang F, Bai J, Zeng XC, Wu H (2015). Compression limit of two-dimensional water constrained in graphene nanocapillaries. ACS Nano.

[30] Chen J, Schusteritsch G, Pickard CJ, Salzmann CG, Michaelides A (2016). Two dimensional ice from first principles: Structures and phase transitions. Phys. Rev. Lett..

[31] Corsetti F, Matthews P, Artacho E (2016). Structural and configurational properties of nanoconfined monolayer ice from first principles. Sci. Rep.

[32] Corsetti F, Zubeltzu J, Artacho E (2016). Enhanced configurational entropy in high-density nanoconfined bilayer ice. Phys. Rev. Lett..

[33] Shih C-J, Lin S, Sharma R, Strano MS, Blankschtein D (2011). Understanding the ph-dependent behavior of graphene oxide aqueous solutions: A comparative experimental and molecular dynamics simulation study. Langmuir.

[34] Tummala NR, Striolo A (2008). Role of counterion condensation in the self-assembly of sds surfactants at the water-graphite interface. J. Phys. Chem. B.

[35] Wei N, Lv C, Xu Z (2014). Wetting of graphene oxide: A molecular dynamics study. Langmuir.

[36] Samadashvili N, Reischl B, Hynninen T, Ala-Nissilä T, Foster AS (2013). Atomistic simulations of friction at an ice-ice interface. Friction.

[37] Giovambattista N, Rossky PJ, Debenedetti PG (2009). Phase transitions induced by nanoconfinement in liquid water. Phys. Rev. Lett..

[38] Vega C, Abascal JLF (2011). Simulating water with rigid non-polarizable models: A general perspective. Phys. Chem. Chem. Phys..

[39] David P, Haitao L (2015). Wettability of graphene. 2D Mater.

[40] Wu Y, Aluru N (2013). Graphitic carbon-water nonbonded interaction parameters. J. Phys. Chem. B.

[41] Leroy F, Liu S, Zhang J (2015). Parametrizing nonbonded interactions from wetting experiments via the work of adhesion: Example of water on graphene surfaces. J. Phys. Chem. C.

[42] Rafiee J (2012). Wetting transparency of graphene. Nat. Mater..

[43] Gurarslan A (2016). Van der waals force isolation of monolayer MoS_2_. Adv. Mater..

[44] Hockney, R. W. & Eastwood, J. W. *Computer Simulation using Particles* (Taylor & Francis, 1989).

[45] Allen, M. P. & Tildesley, D. J. *Computer Simulation of Liquids* (Oxford university press, 1989).

[46] Martí J, Padro JA, Guàrdia E (1996). Molecular dynamics simulation of liquid water along the coexistence curve: Hydrogen bonds and vibrational spectra. J. Chem. Phys..

[47] Martí J (1999). Analysis of the hydrogen bonding and vibrational spectra of supercritical model water by molecular dynamics simulations. J. Chem. Phys..

[48] Falk K, Sedlmeier F, Joly L, Netz RR, Bocquet L (2010). Molecular origin of fast water transport in carbon nanotube membranes: Superlubricity versus curvature dependent friction. Nano Lett..

