# Supplementary material for: Structures and thermodynamics of water encapsulated by graphene
Source: Sci Rep. 2017 Jun 1;7:2646. doi: 10.1038/s41598-017-02582-7 (PMC5453971; doi:10.1038/s41598-017-02582-7)
Supplement: Supplementary file 1 — Supplementary Information Material [file 41598_2017_2582_MOESM1_ESM.pdf]

Supplementary Information for

Structures and Thermodynamics of Water Encapsulated by Graphene

Shuping Jiao[1], Chuanhua Duan[2] and Zhiping Xu[1,*]

[1]Applied Mechanics Laboratory, Department of Engineering Mechanics, and Center for Nano and Micro Mechanics, Tsinghua University, Beijing 100084, China

[2]Department of Mechanical Engineering, Boston University, Boston, MA 02215, USA

[*]Email: xuzp@tsinghua.edu.cn

This Supplementary Information Material contains

- Supplementary Figures S1-S6, Table S1 and Captions

Supplementary Figures, Table and Captions

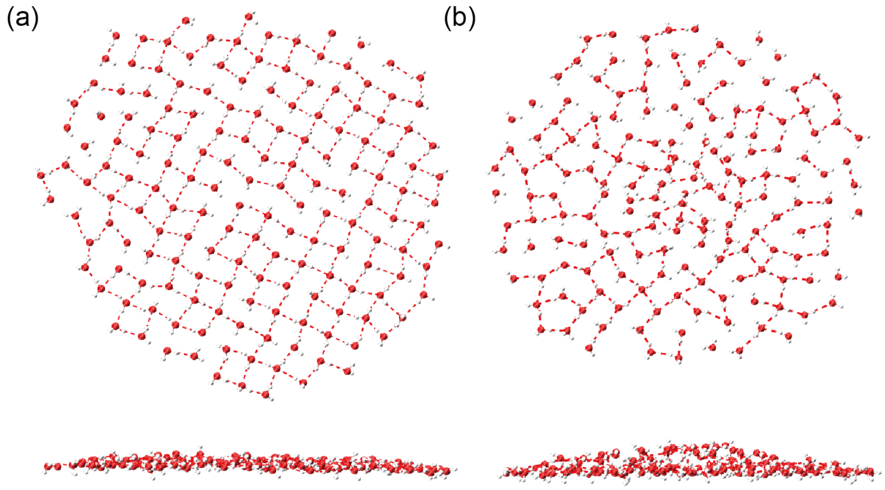

FIG. S1. Snapshots (top and side views) of simulated encapsulated water (EW) with N_W = 165 at (a) T = 400 K and (b) T = 450 K, in the 3D cover model.

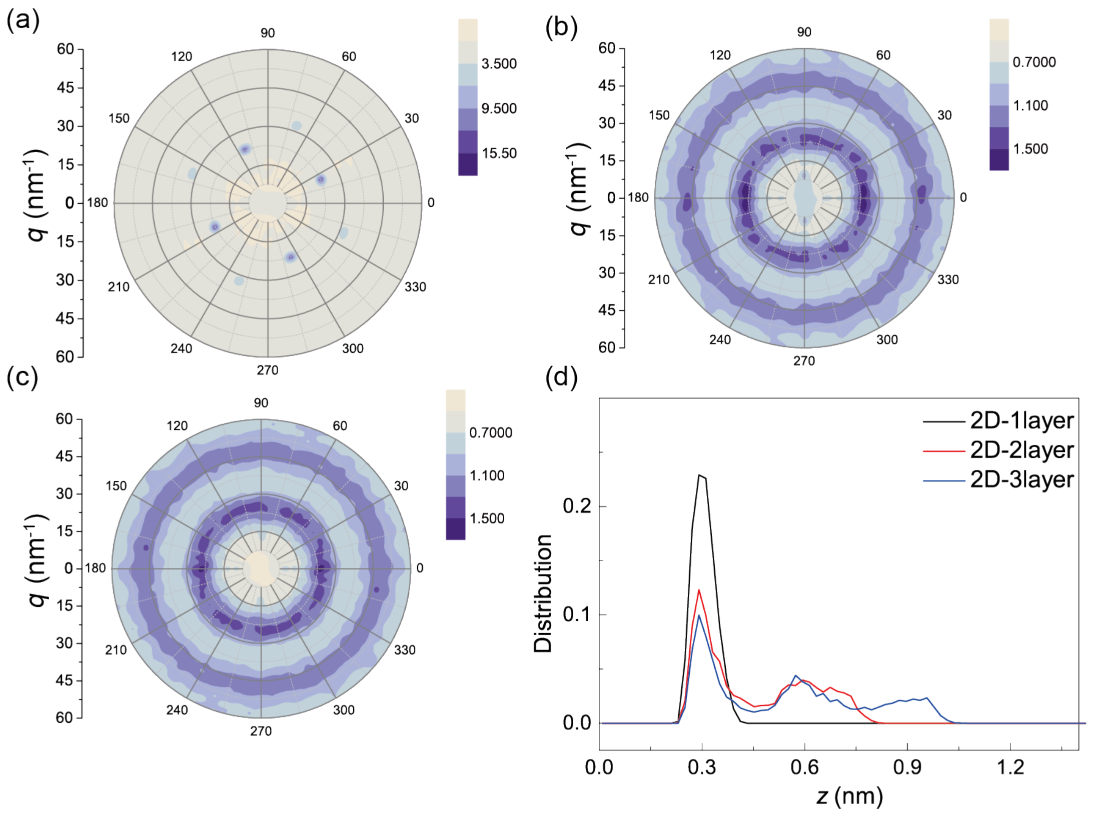

FIG. S2. (a-c) Structure factors of mono-, bi- and tri-layer EW, with the number of water molecules N_W = 60, 181, 354 at T = 300 K. (d) The z-direction density profile of 2D (half-cylindrical) EWs with mono-, bi- and tri-layer structures, at T = 300 K.

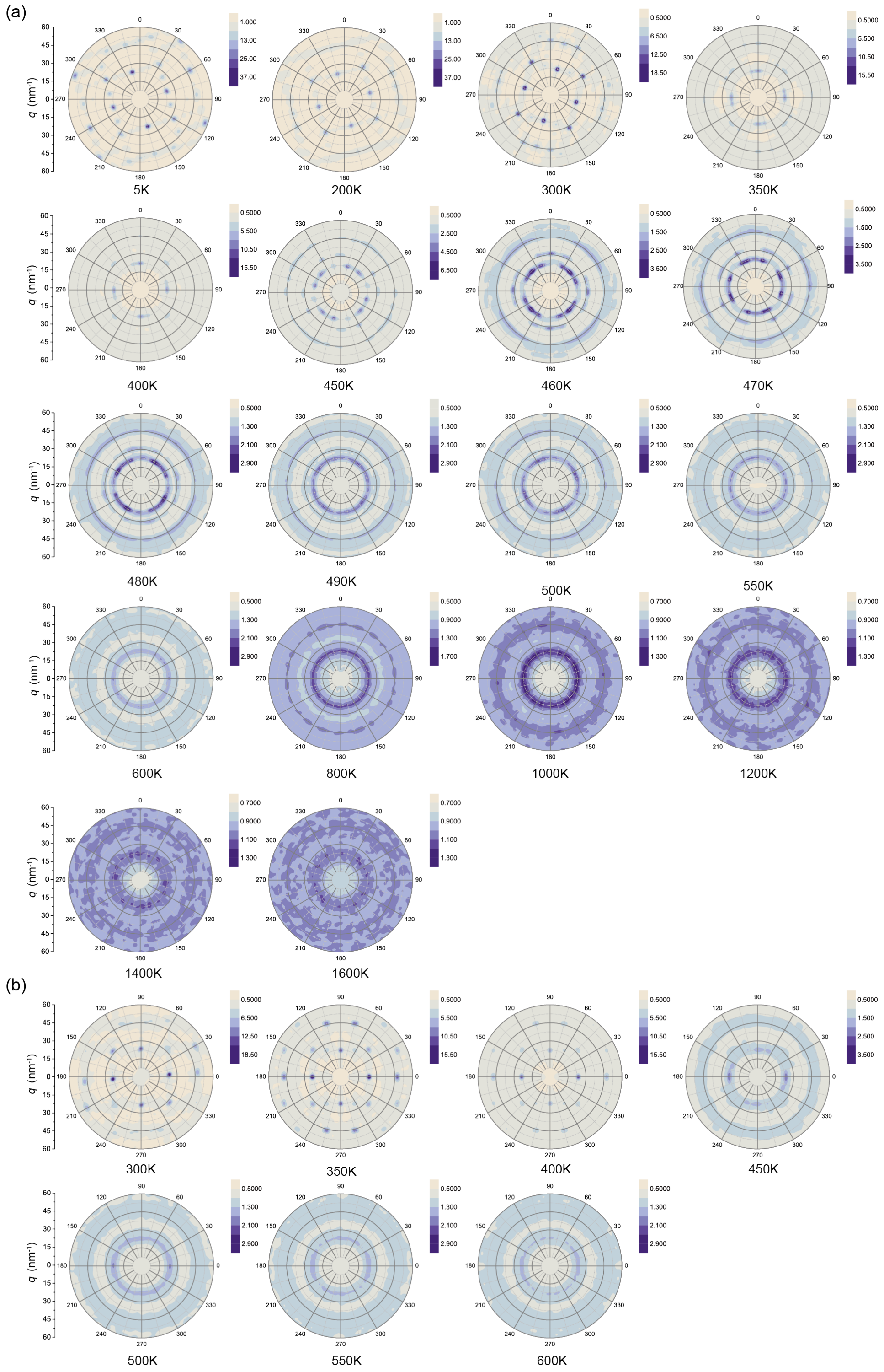

FIG. S3. Structure factors of monolayer EW (N_W = 115) at different temperature in the 3D (hemispheric) (a) and 2D (b) structure.

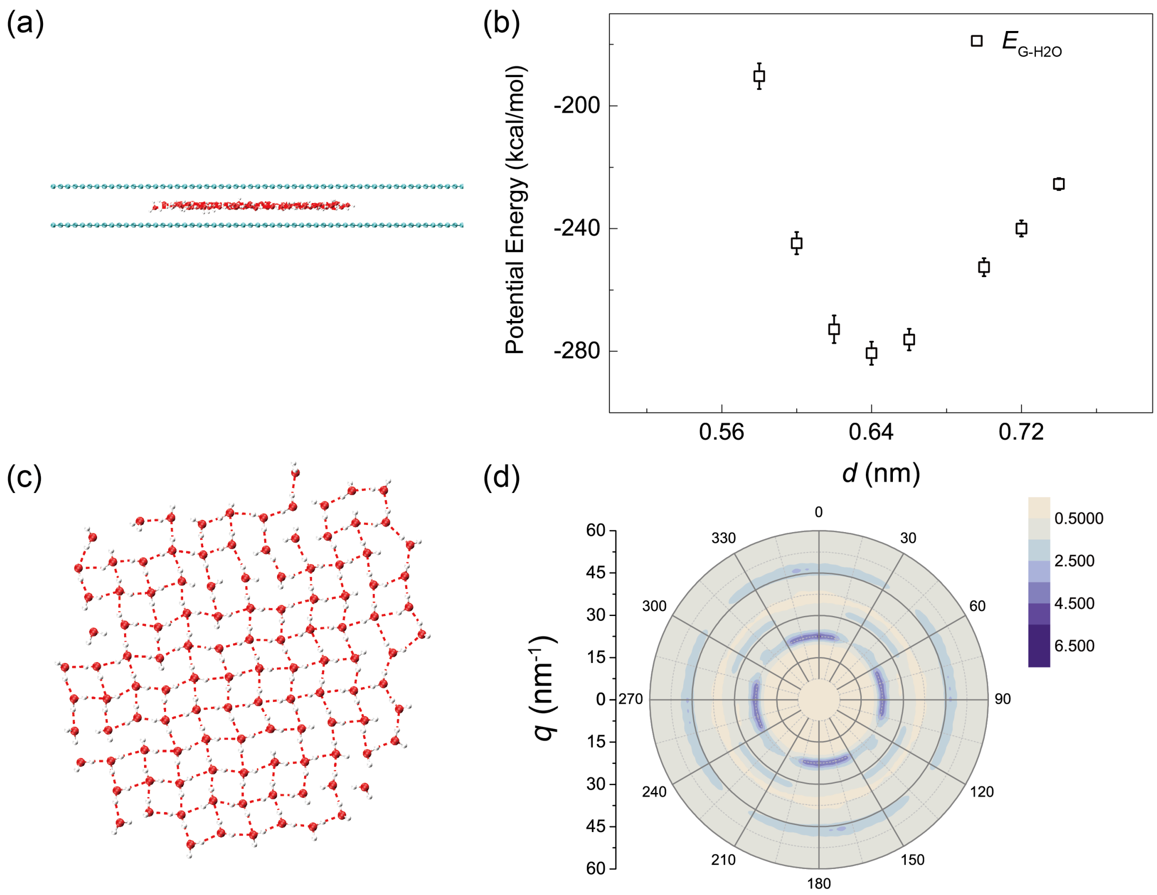

FIG. S4. (a) The atomistic model with intercalated water (N_W = 115) between two flat graphene sheets with an interlayer distance d, at 300 K. (b) The potential energy of intercalated water as a function of d, with a minimum at d = 0.64 nm. (c, d) The atomic structures and structure factor of the intercalated water structures at d = 0.64 nm, and T = 300 K.

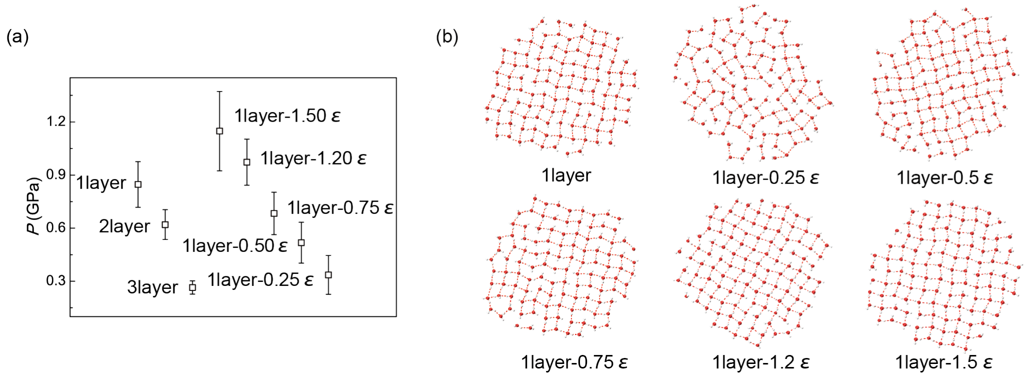

FIG. S5. (a) The pressure in mono-, bi- and tri-layer EW structures (N_W = 115, T = 300 K) under 3D graphene cover, calculated using the Lennard-Jones potential parameter ε and several modified values (0.25ε, 0.5ε, 0.75ε, 1.2ε, 1.5ε). The reference value ε is parametrized by fitting the water contact angle (WCA) of graphene to 98°. The WCAs for 1.2ε is 75°. (b) Atomic structures of monolayer EWs predicted by these modified values of ε (N_W = 115, T = 300 K).

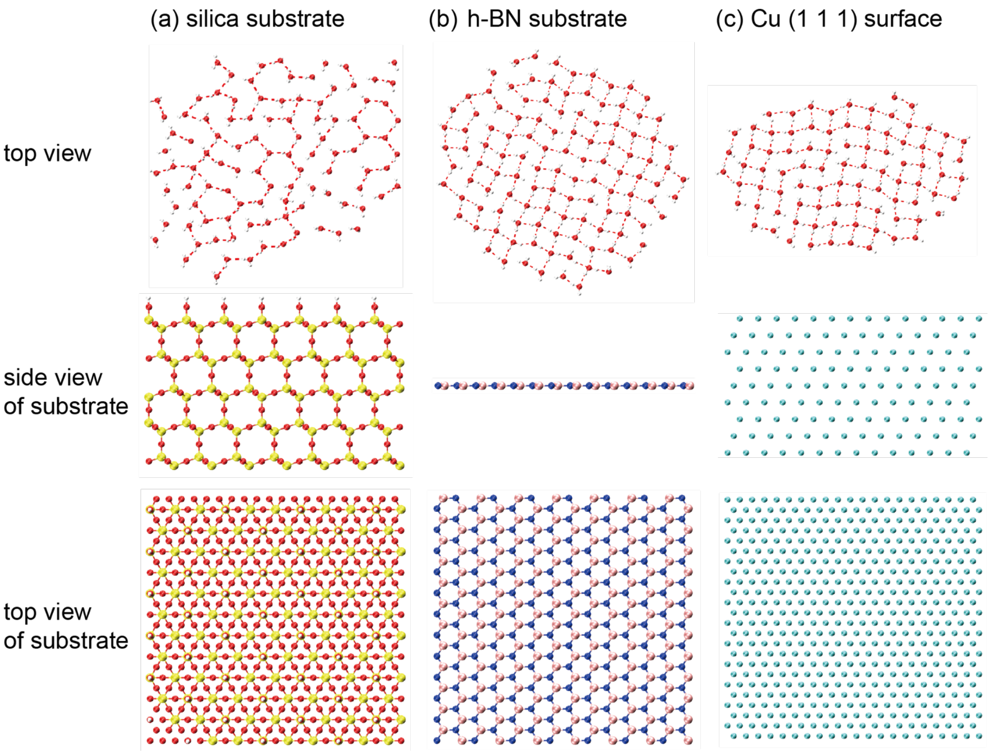

FIG. S6. Snapshots of monolayer EWs confined between graphene and (a) silica and (b) h-BN (c) copper substrate (N_W = 115, T = 300 K) in the 3D cover model. The forcefield parameters, including atomic charges and interatomic interaction parameters, are taken from Refs.[1-4]

Table S1. Lennard-Jones and atomic-charge parameters used for SiO_2, BN, Cu substrates for the results summarized in **Fig. S6**.[1-4]

Atom	σ (Kcal/mol)	ε (Å)	Atomic charge (e)
Si	0	3.302	2.1
BO[1] (in SiO_2)	0.15533	3.166	-1.05
SO[2] (in SiO_2)	0.15533	3.166	-0.95
H (in SiO_2)	0	0	0.425
B	0.09486	3.453	0.93
N	0.14477	3.365	-0.93
Cu	3.85	2.314	0

[1]BO: bridge oxygen atoms in SiO_2, [2]SO: oxygen atoms in the surface hydroxyl of silica. Lorentz-Berthelot mixing rules are used for atomic pairs between different species.

References

1 Dutta, R. C., Khan, S. & Singh, J. K. Wetting transition of water on graphite and boron-nitride surfaces: A molecular dynamics study. Fluid Phase Equilib. 302, 310-315 (2011).

2 Ho, T. A. et al. Interfacial water on crystalline silica: a comparative molecular dynamics simulation study. Mol.Simul. 37, 172-195 (2011).

3 Li, H. & Zeng, X. C. Wetting and interfacial properties of water nanodroplets in contact with graphene and monolayer boron-nitride sheets. ACS Nano 6, 2401-2409 (2012).

4 Wang, Y. & Xu, Z. Water Intercalation for Seamless, Electrically Insulating, and Thermally Transparent Interfaces. ACS Appl. Mater. Interfaces 8, 1970-1976 (2016).
